# Balancing Freedom of Expression and Social Responsibility on the Internet

**DOI:** 10.1007/s11406-017-9856-6

**Published:** 2017-06-06

**Authors:** Raphael Cohen-Almagor

**Affiliations:** School of Law and Politics, The University of Hull, Cottingham Road, Hull, HU6 7RX, UK

**Keywords:** Business ethics, Hate speech, Internet gatekeepers, Responsibility, Terror

## Abstract

This paper reflects on the articles submitted for the Symposium *Confronting the Internet’s Dark Side.* I discuss some of the criticisms of the book’s theory and my treatment of hate speech. The responsibilities of Internet Service Providers (ISPs) and Web-Hosting Services (WHSs) are in the fore, arguing that as they are the gatekeepers, they need to be proactive far more than they are now. This paper, like my book, strives to suggest an approach that harnesses the strengths and capabilities of the public and the private sectors in offering practical solutions to pressing problems.

## Introduction

The Internet’s design and raison d’être are complete freedom, but soon enough people began to exploit the Net’s massive potential to enhance partisan interests, some of which are harmful and anti-social. Given that the Internet has been part of our lives for a relatively short time, the discussions about the costs and harms of the Internet, and how to address them, are in their infancy. The transnational nature of the World-Wide-Web makes regulation very difficult, if not impossible.

The articles in this volume reflect on some aspects of the theory invoked in of *Confronting the Internet’s Dark Side,* on the role of Internet intermediaries, and on the lingering problem of hate speech. In this paper, I intend to critically reflect on some of the arguments and to expand on the book’s reasoning.

## Theory

In his article, “Business Ethics and Free Speech on the Internet”, Brian Berkey provides much food for thought. He seems to accept that John Stuart Mill’s theory can be a good starting point to discuss freedom of expression on the Internet. He wants to deduce from Mill whether Internet Service Providers (ISPs) and Web-Hosting Services (WHSs) ought to deny service to those who would engage in online hate speech. But he takes Mill’s position too literary with not enough consideration to the dramatic technological and media developments that have taken place since Mill wrote *On Liberty* in 1869. In another article (Cohen-Almagor 2012) I explained that Mill wrote at a time when there was no mass media. All there was were a few newspapers, read only by the elite. Today the situation is very different. People have access to newspapers, radio, television, the Internet, cell phones, and other technological means of communication that together influence and shape to one extent or another the reality in which we live. Together these means of mass communication can create and reinforce an atmosphere of incitement. Dozens of calls for hatred and violence might create a turbulent atmosphere that is conducive to violence. Frequently, inciting speech, even if repeated and magnified by the media, falls on deaf ears. Sometimes, however, such speech falls on eager ears and can be a factor in inducing the already-persuaded that there is a need to take action; i.e., to move from harmful speech to harmful deeds. It is one thing to throw a lighted cigarette into an empty bin and quite another to throw it in a dry field. As Zechariah Chafee (1946: 397) stated: “Smoking is all right, but not in a powder magazine.”

## The Role of Internet Intermediaries

The authors of “The Psychology of Social Networking: The Challenges of Social Networking for Fame-Valuing Teens' Body Image” argue that social networking seems to satisfy some adolescent developmental needs. First, it allows teens to practice identity establishment through performing self-expression and self-presentation tasks. Second, it provides opportunities for youth to seek social approval from others. Furthermore, adolescents have a strong attraction and aspiration to fame, which has become one of the most highly appreciated values for this age group. Tali Te’enu-Harari and Keren Eyal conclude that youth’s exposure to thin-idealizing content posted by their adored celebrities on interactive and highly engaging social networking sites poses potential challenges for these young, developing individuals and therefore social networking sites should adopt basic norms of moral and social responsibility. This recommendation is hammered time and again in *Confronting the Internet’s Dark Side.*

In my book, I argued that Internet intermediaries should not be equated with telephone service providers or with electric service providers. I explained the major technical differences as well as differences in scope and potential impact. Professor Berkey criticises me for failing to explain why any of these differences are morally important. I should have made the point clearer. The technological differences are of functional, practical importance. The possibilities that the Internet is offering exceed by far those that are made available by the telephone providers. The Internet cannot be seen as mere electric grid. While these differences are not necessarily morally significant, the practical implications of Internet abuse are certainly morally significant. Therefore I insist that Internet intermediaries should be more responsible and more proactive in ensuring safe use of this innovative and transformative information platform.

After refuting the telephone provider and the electric grid analogies, I went on to offer what I believe are better analogies to understand social responsibility on the Internet. I have argued that better analogies are those between the Internet and a large first and second hand bookstore, or between the Internet and a large library. An owner of a bookstore cannot be held responsible for the content of each and every book in her store. She does not read and inspect all the books. Similarly, it can be argued, an Internet provider should not be held accountable for content on its server. But if a bookstore owner is informed that a specific book contains child pornography, some other illegal material, or material that violates copyright, and she does not take the book off the shelves, then the owner may be held legally responsible for violation of the law. And she is also morally responsible. Similarly, it can be argued, is the case on the Internet. ISPs and WHSs have discretion whether their services are opened for all or limited in one way or another.

Bookstore owners do have discretion about the books they offer for buyers. Many would like to maintain a quiet and tranquil atmosphere in the store. The books, accordingly, will be for the general readership. Other book owners might opt for a more rowdy atmosphere. They will entertain books of socially problematic material. The likely result would be that the general readership would refrain from visiting those bookstores. Those stores would become niche stores, for particular readers.

In both kinds of store, their owners would like to keep the business going. They will listen to warnings about the illegality of certain books. The same, it can be argued, is true of ISPs and WHSs: Provide a notice first, allowing the provider to make a decision about the consequences to which she might be held liable. If the provider/host does not act upon the warning, then it will have to face the consequences. Indeed, on copyrights issues ISPs are expected to assume responsibility. They should also assume moral and social responsibility when violent, anti-social activities are taking place on their servers. Most ISPs and web-hosting companies would not like their servers to be transformed into forums in which people concoct criminal activities.

Professor Berkey offers several criticisms with regard to this analogy. First, the discretion that bookstore owners is quite wide, wider than what he would like Internet intermediaries to have. For instance, bookstore owners may operate religious stores that sell only books of one religion or they may opt to sell only books of a certain ideological leaning. I would certainly not wish to see ISPs that adapt only one conception of the good. Having said that, I did mention in my book the possibility of niche bookstores with the immediate implications of being a niche.

Professor Berkey further argues that in my view Internet intermediaries are permitted to discriminate only against hate speech. This is incorrect. Throughout my book, I argued that ISPs and WHSs should adopt responsible standards of conduct to protect Netusers from the harms of cyber bullying, terror, crime and hate speech.

Internet Service Providers (ISPs) and Web-Hosting Services (WHSs) have become major actors in shaping the informational environment and in influencing users’ experiences and interactions within it. They provide open infrastructure and applications that facilitate digital expression, interaction, and the communication of information. I argue that Internet intermediaries have social responsibility to the public they serve. They are trustees of the greater good. Gatekeeping entails promoting of an ethical and responsible informational environment in which users can surf the Internet safely and in a way that support the common good.

In August 2016, the UK Home Affairs Committee published a report on extremism which affects Muslim communities and arising from the activities of terrorist organisations such as Daesh. The report recommends that the government adopt a sophisticated approach both to identifying the factors which instigate radicalisation and in the measures it takes to tackle radicalisation. The report includes many detailed recommendations about the work of the Metropolitan Police’s Counter Terrorism Internet Referral Unit (CTIRU) and the Europol, the PREVENT strategy and border security; it probes the role of communities, media and technology, aiming to contain radicalization and promote security and peace of mind (House of Commons Home Affairs Committee 2016).

When discussing the role of the Internet and social media in facilitating and encouraging terror, the reiterated theme in the report is responsibility. The fight against radicalization and terror requires cooperation of all pertinent stakeholders and shared responsibility to take adequate measures to prevent violence.

The report holds that “Social media companies are consciously failing to combat the use of their sites to promote terrorism and killings. Networks like Facebook, Twitter and YouTube are the vehicle of choice in spreading propaganda and they have become the recruiting platforms for terrorism. They must accept that the hundreds of millions in revenues generated from billions of people using their products needs to be accompanied by a greater sense of responsibility and ownership for the impact that extremist material on their sites is having” (House of Commons Home Affairs Committee 2016). The report calls for a zero tolerance approach to online extremism, including enticement to join extremist groups or commit attacks of terror and any glorification of such activities. The report recommends that manuals for terrorists and extremists should be removed from the Internet.

The report voiced dismay that social media companies have teams of only a few hundred employees to monitor networks of billions of accounts and that Twitter does not even proactively report extremist content to law enforcement agencies. The report says that “these companies are hiding behind their supranational legal status to pass the parcel of responsibility and refusing to act responsibly in case they damage their brands. If they continue to fail to tackle this issue and allow their platforms to become the ‘Wild West’ of the Internet, then it will erode their reputation as responsible operators” (House of Commons Home Affairs Committee 2016).

These are bold and correct statements. The only reservation I have is with regard to the claim that their conduct “will erode their reputation as responsible operators”. I do not think that these companies have tried to gain such a reputation and consequently they were never regarded as responsible by people who have been studying political extremism and terror on the Internet. During the past decade I spoke with dozens of security officers in the UK, the USA, Canada, Australia and Israel. They all voiced their growing frustration with the free speech, neutral attitude adopted by the media giants.

### Twitter

has the most expansive boundaries to freedom of expression. Its terms of service state that “All Content, whether publicly posted or privately transmitted, is the sole responsibility of the person who originated such Content. We may not monitor or control the Content posted via the Services and, we cannot take responsibility for such Content. Any use or reliance on any Content or materials posted via the Services or obtained by you through the Services is at your own risk”.^1^ Twitter does have some rules that prescribe content in limited circumstances.^2^ Holocaust denial and, more broadly hate speech do not count among those circumstances.

### YouTube

community guidelines include reference to hateful content. It says:

## Hateful Content

Our products are platforms for free expression. But we don't support content that promotes or condones violence against individuals or groups based on race or ethnic origin, religion, disability, gender, age, nationality, veteran status, or sexual orientation/gender identity, or whose primary purpose is inciting hatred on the basis of these core characteristics. This can be a delicate balancing act, but if the primary purpose is to attack a protected group, the content crosses the line.^3^ Yet on YouTube one may find “The ‘Holohoax’ exposed in 30 minutes”^4^ and a number of other “Holhoax” videos, including the Leuchter Report^5^ and a string of 23 clips whose common theme is “no single Jew died in a gas chamber”.^6^

### Facebook

prohibits posting content that is hateful or threatening. Facebook disabled a group called ‘I Hate Muslims in Oz.’ Barry Schnitt explained: “We disabled the ‘I Hate Muslims in Oz’ group… because it contained an explicit statement of hate. Where Holocaust-denial groups have done this and been reported, we’ve taken the same action” (Matyszczyk 2009). In May 2010, Facebook took down a page titled “Kill a Jew Day,” which urged Netusers to violence “anywhere you see a Jew” between July 4 and July 22.

### Facebook

distinguishes between ‘explicit statement of hate’ and Holocaust denial. Its directors believe that Holocaust denial is not hateful per se and does not therefore contravene the company’s terms of service. The terms of service say: “You will not post content that is hateful, threatening, pornographic, or that contains nudity or graphic or gratuitous violence”.^7^ Facebook still hosts the National Association for the Advancement of White People.^8^ Facebook said that “We think it's important to maintain consistency in our policies, which don't generally prohibit people from making statements about historical events, no matter how ignorant the statement or how awful the event” (Grossman 2011). How can this stance be reconciled with Facebook prohibition on posting content that is hateful or threatening is something for the Facebook managers to reckon with and answer.

## Hate Speech

Hate speech is a contested topic and the extent that it is debated is exemplified in the articles in this symposium. While Brian Berkey thinks that my intolerance of hate speech is exaggerated, Bhikhu Parekh is inclined to go further than me and insist on not tolerating the bigot at all. I shall first avail myself to discuss Parekh’s critique and then move on to consider Professor Berkey’s arguments.

Lord Professor Parekh argues that hate speech is objectionable for both intrinsic and instrumental reasons. He counts the various harms that hate speech inflicts on its targets and rightly explains that the danger of hate speech should be assessed against the prevailing social climate and the lessons from history. Parekh brings the example of a radical group that urges people to kill all elderly parents or all beautiful women. Its intended audience as well as well as its target group would consider this urging as mad or as a poor joke and would dismiss this utterance. But if similar sentiments are expressed about blacks, Jews, or gays, then they would be seriously considered. This is because of the deeply rooted prejudices against these groups, and because the lessons we have learned from history.

Lord Parekh is right. We assess the legitimacy of expressions in accordance with social and historical data. To give another example: If someone were to express the thought: Jews should be gassed during the 1920s, the utterance would be dismissed as ridiculous, crazy and also impractical thought. What does it mean gas all the Jews? How is this possible? Even if the audience were to be deeply anti-Semitic, they would still question the validity and practicality of this suggestion to gas all Jews.

Today, after the horror of the holocaust, the same statement is likely to evoke strong negative public reaction. The assertion would no longer be dismissed as mad but would be taken most seriously. We do learn some lessons.

Lord Parekh speaks in support of a law that would ban hate speech. I addressed the issue in my 2012 article and find myself in much agreement with my good friend Bhikhu. Like him, I think that we have to fight against hate and bigotry. Like him, I think that hate speech has no place in a decent society and deserves to be discouraged. Like him I believe there is room to invoke restrictive laws as last resort, after exhausting educational, social and moral means. Parekh and I (and also Waldron 2012) believe that hate speech legislation should protect people’s dignity against assault. It should aim to protect the targets’ equal status in the community, their entitlement to basic justice and to the fundamentals of their reputation. Parekh and I speak of maintaining civility and human dignity. A person’s dignity relates to people’s social standing, the fundamentals of basic reputation that entitle them to be treated as equals in society. Hate speech aims to undermine its target’s reputation by associating characteristics like race, ethnicity or religion with attributes that would disqualify them from enjoying equal standing in society. Hate speech undercut the targets’ entitlement to basic justice and to the fundamentals of their reputation. As students of history, it would be utterly irresponsible on our part to turn a blind eye, legitimize or condone in one way or another the existence of such discriminatory, indeed evil talk in society. As Parekh rightly notes, we may differ in our view as to when is time to invoke such laws. We both use the term “last resort” but we may differ in our understanding when the time comes to see the necessity in such laws. It seems to me that Parekh would see the necessity of such laws in an earlier stage. When hate speech is concerned, he would be inclined to weigh freedom of expression less favourably than the competing interests of protecting minorities and maintaining civility.

Lord Parekh and I share concerns regarding the marketplace of ideas concept. Both of us are cognizant of its limitations.^9^ Parekh writes that he is “a little troubled” by my “heavy reliance on the Millian Principle of interfering with an individual only when his or her utterances and actions threaten to cause harm to others”. This is because this view puts the onus not on the speaker but on his target group, and in so doing places the latter at disadvantage. Second, because harm is not easy to define, identify and prove. He says that I am right to insist that “exaggerated and ill-focused remarks about harm should not be made the basis of actions on the internet or elsewhere, but the opposite extreme of requiring a fairly precise casual connection between a particular form of hate speech and a particular kind of harm would not do either”. It seems that Parekh sees no value in being tolerant to hate speech. He does not think that such restrictions would go against the spirit of democracy. Quite the opposite. Restrictions on hate speech would benefit democracy and society.

Professor Berkey, on the other hand, argues that my definition of hate speech is overly broad though in one sense it is curiously narrow. In my book, hate speech is defined as a bias-motivated, hostile, malicious speech aimed at a person or a group of people because of some of their actual or perceived innate characteristics. It expresses discriminatory, intimidating, disapproving, antagonistic and/or prejudicial attitudes toward the disliked target group (Cohen-Almagor 2015). Berkey thinks that this definition is broad because a great deal of speech is bias motivated, hostile and malicious, and not all of it amounts to hate speech. In his example, Jane watches a video featuring an African American artist performing a violent song. Jane, who is a white woman, does not like it and she posts online comment saying that the artist should be in prison. Professor Berkey rightly says that this comment is bias motivated, hostile and malicious but it is not hate speech. I agree with Berkey. It is not hate speech because it is not aimed at the artist due to his actual or perceived innate characteristics.

Professor Berkey then considers Jim who posts the following comment: “Scientific evidence shows that members of racial group A are naturally more intelligent than members of racial group B”. The statement might be morally repugnant but, Berkey argues, it does not amount hate speech. Now this is an interesting example.

For many years, I have been presenting to my students the case of Jean-Philippe Rushton, a Canadian psychology professor who has argued about hierarchy of races: Asians are smarter than whites, who are in turn smarter than blacks. In his book *Race, Evolution, and Behavior* (1999) Rushton explained that brain and genital size are inversely related, and that races differ in brain size, intelligence, sexual behaviour, fertility, personality, maturation, lifespan, crime and in family stability. He explained that blacks are less intelligent than Orientals and Whites and they are more involved in criminal activities. While the IQ of Orientals is about 106, the IQ of Black people is around 70 to 75. Black people are also more sexually promiscuous and they lack social organization. Here are some quotes from his book:

On average, Orientals are slower to mature, less fertile and less sexually active, have larger brains and higher IQ scores. Blacks are at the opposite end in each of these areas. Whites fall in the middle, often close to Orientals (p. 18).

Blacks are more aggressive and outgoing than Whites, while Whites are more aggressive and outgoing than Orientals. Blacks also have more mental instability than Whites. Black rates of drug and alcohol abuse are higher (pp. 33-34).

Orientals have about 102 million more brain cells than Whites, and… Whites have about 480 million more than Blacks. These differences in brain size probably explain the racial differences in IQ and cultural achievement (p. 58).

… the difference in average crime rate means that a much higher percentage of Blacks fall into a life of crime. The 85 average IQ of criminals is almost identical with the 85 average IQ of Blacks, so IQ is related to crime (pp. 94-95).

The science behind these assertions is debatable. Rushton’s theory evoked much criticism and has been perceived as racist (Zuckerman and Brody 1988; Anderson 1991; Allemang 2012). His theory attempts to explain everything by the sole criterion of race. It ignores social circumstances and social construction. It does not take into account other, no less important factors, such as individual abilities, class, poverty, education and family infrastructure. But is it hate speech? In the spirit of the liberal marketplace of ideas, the search for the truth and open disputation of ideas with contrasting ideas, one may think that Rushton’s theory is problematic but it should be tolerated and debated. Its scientific facade needs to be exposed and simultaneously the true motives that guide Rushton should be explored. This, indeed, is my belief. Rushton’s theory is a hard case. It is opened to interpretations but it should not be silenced.

I also believe that Rushton’s theory was not guided only by scientific methods, that it had underpinning agenda which was not innocent, that it was motivated by other reasons rather than the urge to discover a scientific truth. Rushton (1999, 104) was asked “Weren’t theories about race differences the reason for racism, genocide and the Holocaust?” Rushton answered (1999, 104-105): “The Nazis and others used their supposed racial superiority to justify war and genocide. But just about every idea - nationalism, religion, egalitarianism, even self-defence - has been used as an excuse for war, oppression or genocide. Science, however, is objective. It can’t give us our goals, but it can tell us how easy or difficult it will be to reach our goal. Knowing more about race differences may help us to give every child the best possible education and help us to understand some of our chronic social problems better”.

With this answer, Rushton was trivializing the Nazi crimes. Nazism was equated with nationalism, religion, egalitarianism, “even self-defence”. Rushton says nothing about the evil ideas of Nazism per se but how they were used for evil deeds, in the same way that other ideas, including noble ideas such as egalitarianism and well-established ideas such as self-defence, have been used for evil deeds. Then Rushton declares that his science is objective. His commitment is to scientific truth, no matter how crude that truth might be. And then he goes on to argue that his ideas may better children education. But surely not the education of *every* child. No matter how much you invest in the education of black children, they would not be able to escape their lot. They belong to the inferior race and therefore they are doomed to suffer the consequences of their brute luck.

What can help us understand Rushton’s reasoning is his behaviour and conduct outside the scientific world. Rushton was embraced by anti-black associations, by racists and bigots. Rushton not only did not flinch; he accepted their attention and the honour of being their star scientist. In 2002, Rushton was appointed president of the Pioneer Fund, which has for decades funded dubious studies linking race to characteristics like criminality, sexuality and intelligence. Pioneer has long promoted eugenics, or the “science” of creating “better” humans through selective breeding. Set up in 1937 and headed by Nazi sympathizers, the Pioneer Fund’s mission was "to advance the scientific study of heredity and human differences”.^10^ It strove to improve the character of the American people through eugenics and procreation by people of white colonial stock. Rushton has spoken on the alleged IQ deficiencies of minorities at conferences of the racist *American Renaissance* magazine and website, and he has published a number of articles in the group’s newsletter. His work is often published on racist websites, including the anti-immigrant hate site, Vdare.com.^11^ While appearing before and in support of racist groups, the above-mentioned sensitive and debatable statements then amount to hate speech. The context, as we learned from JS Mill’s theory on liberty (1948), makes a great difference. A questionable race theory when invoked in radical extremist rallies is the fuel for their raging hatred, the validating force for their twisted beliefs, the scientific cloth that legitimized crude beliefs about hierarchy of races. Expressed in such forums, Rushton’s ideas become hate speech.

Professor Berkey maintains that my definition of hate speech appears to exclude speech that intuitively should count as hate speech. This is because of the requirement that the speech in question is directed against individuals or groups because of actual or perceived innate characteristics. Berkey understands that as a result hate speech against religious groups cannot be defined as hate speech. By innate characteristics I meant a quality or ability that one is born with to be distinguished from a quality or ability one that have learned. Thus hateful speech against religious people would certainly fall within the definition of hate speech. True, people are free to change their religion. They also free to change their sex and/or nationality, as some indeed do. But they are unable to change the past.

Hate speech is harmful and dangerous. It may have, indeed it has, significant effect on the lives of targeted groups. In *Confronting the Internet’s Dark Side* I emphasised the connection between hate speech and hate crime. Bigots, inspired by what they have read online, went on to inflict violence on their targets. I am not going to repeat the evidence provided in the book. Here I want to highlight the connection between hate groups and biochemical warfare. Hate groups have talked for years about using anthrax to strike at the U.S. government. Chemical and biological warfare can result in many casualties, an attractive proposition for hate mongers who opt for harmful action. In 1995, Larry Wayne Harris, a microbiologist and former member of the Aryan Nations, was arrested in Ohio with three vials of bubonic plague toxin he had ordered fraudulently by mail from a supplier in Maryland. He was given 18 months on probation. He wrote "Bacteriological Warfare: A Major Threat to North America,” which may be considered a how-to book. The FBI found legal veterinary anthrax vaccine in the trunk of his car (Slevin 2001a, b).

The same year, 1995, members of the Patriot’s Council were arrested in Minnesota and charged with manufacturing ricin, a deadly biochemical substance, to kill law enforcement officers. In 1998, members of a Texas anti-government group were charged with plotting to infect people with cactus needles dipped in anthrax or the AIDS virus (Slevin 2001a, b). Alexander James Curtis, arrested in 2001 in San Diego on charges of harassing civil rights leaders and vandalizing two synagogues, published an Internet guide in 2000 called “Biology for Aryans” that described the use of botulism, anthrax and typhoid for terror. Curtis had extensive Internet connections in which he kept in touch with like-minded individuals and had spread his message of intolerance nationwide (Perry 2000). Curtis also published the monthly Nationalist Observer on the Internet and offered a weekly and daily telephone broadcast and a racist Internet magazine in which he advocated biological terrorism and celebrated “lone wolves” such as Timothy McVeigh and Buford Furrow. Indicted with Curtis were three men who met him through his extensive websites dedicated to preaching racial superiority and violence: Michael Brian DaSilva, 21; Robert Nicol Morehouse, 53; and Kevin Christopher Holland, 22. The four defendants were charged with violating federal civil rights and hate-crime laws that make it illegal to target someone for mistreatment on the basis of race, religion or national origin (Perry and Murphy 2000; Web-Based White Supremacist and followers charged with hate crimes 2000).

Other dangerous publications are “Biotoxic Weapons” and “Advanced Biological Weapons Design and Manufacture.” These are virtual cookbooks for anthrax and other biological weapons. Each states it is “for academic purposes only.” The author, Timothy Tobiason, was selling his self-published books and compact discs at gun shows for years. Raymond A. Zilinskas, a senior scientist at the Center for Nonproliferation Studies in Monterey, California., said, “he does give some accurate information on how to process spores that I have not seen anywhere else in the open literature’ (Thomas 2001). In brackets I should mention that cryptome.org posts in the name of freedom of information dangerous material, including a detailed patent of ricin.^12^ And the Los Alamos Primer published documents of the energy department containing what is needed in order to produce an atomic bomb (Serber 1992). This is protected speech under the First Amendment. In this era of global terrorism and nuclear proliferation, I question whether this is also a responsible speech.

While my approach prefers adopting standards of civility and social responsibility by all stakeholders – Netusers who upload information onto the Net, readers, Internet gatekeepers, countries and the international community at large, and while I see the need for legislation as a last resort after exhausting all other methods short of legislation (education, open debate, deliberative procedures facilitate by the media, discussions and consultations with the Internet industry), Amos N. Guiora argues for legislation. Given the demonstrated impact of social media on our daily lives, Guiora contends that a values–based approach must be buttressed by legal standards and limits. Adopting and re-articulation the American *Brandenburg* test,^13^ Guiora calls for limiting social media speech because we can no longer afford to ignore the harm posed by Internet hate speech. Postings on social media must be assessed on a sliding scale taking into account multiple factors including magnitude, frequency, intent of the platform, and content of the post and platform.

## Conclusion

Why do people differ in their view of such balancing? We are all products of our education and upbringing. We are social beings, influenced by what we witness in our societies, and the values enshrined in our families, significant other group, culture and nation. Freedom of expression is perceived quite differently in the USA, India and in Israel. I think the perception of free speech in Israel is somewhere between India and the USA, probably closer to the American view than to the Indian view.

In his critique of the American First Amendment position, Jeremy Waldron notes (2012, 185) that Britain has laws that prohibit racial and religious hatred (Public Order Act 1986) and racial discrimination (Race Relations Act 1976). Hate speech laws aim to protect the public good of dignity-based assurance, and to block the construction of the rival public good that the racists and Islamophobes are seeking to construct among themselves (Waldron 2012, 95). Are these laws illegitimate? Was their enactment inappropriate and their enforcement morally wrong? Furthermore, almost all democracies have hate speech laws. Are they all wrong and only the United States, which protects hate speech, is right?^14^

My book tries to balance one against the other two important principles: freedom of expression and social responsibility. The forefathers of the Internet had the vision of creating a free highway, a public space where everyone can say what he or she has in mind. This wonderful innovation of unfettered platform has backfired. The Internet is open for use and abuse. We should provide and promote responsible use and we should also fight against those who abuse. The abuse corrupt public space and has posed many challenges on all levels: individual, the community, the state and the international community. We are in the early stages of learning how to cope and how to combat the abuse. Slowly we are developing the necessary tools to enjoy innovation and freedom while, at the same time, we are adopting safeguards and rules of responsible conduct. In my book, I made a distinction between Netusers and Netcitizens. The term “Netuser” refers to people who use the Internet. It is a neutral term. It does not convey any clue as to how people use the Internet. It does not suggest any appraisal of their use.

On the other hand, the term “netcitizen” as it is employed here is not neutral. It describes a responsible use of the Internet. Netcitizens are people who use the Internet as an integral part of their real life. That is to say, their virtual life is not separated from their real life. Even if they invent an identity for themselves on social networks, they do it in a responsible manner. They still hold themselves accountable for the consequences of their Internet use. In other words, netcitizens are good citizens of the Internet. They contribute to the Internet’s use and growth while making an effort to ensure that their communications and Net use are constructive. They foster free speech, open access and social culture of respecting others, and of not harming others. Netcitizens are Netusers with a sense of responsibility.

While a great deal is dependent on how we use the Internet, a great deal is also dependent on the Internet gatekeepers. These companies possess immense power. Sometimes it has been said that Facebook and Google have more power than presidents and prime ministers. I do not think that this statement is exaggerated. Power without responsibility is dangerous. Power without responsibility is corrosive. Power without responsibility undermines our well-being. Therefore we must insist that Internet intermediaries will take responsibility and ensure that Netusers will be able to enjoy the vast capabilities of the Internet without putting themselves in danger. The Internet’s way should not be in harm’s way. The Internet’s way should be enlightening, innovative, entertaining, productive, voicing the best of humanity. To enable this, boundaries should be introduced, antisocial and violent activities should be curbed, safe environment should be established. This is a combined effort of Netusers, business, countries and the international community at large.

## Footnotes

1 Twitter Terms of Service, https://twitter.com/tos

2 The Twitter Rules, https://support.twitter.com/articles/18311-the-twitter-rules

3 Respect the YouTube community, http://www.youtube.com/yt/policyandsafety/communityguidelines.html

4 “The ‘Holohoax’ exposed in 30 min”, https://www.youtube.com/watch?v=bpjqf-vNq6I

5 The Leuchter Report, https://www.youtube.com/watch?v=WLQv_UQ2mJ0&list=PL2zouJfRSfG52G5LVQwLOD6nj4Ji8l7wE

6
https://www.youtube.com/watch?v=CsrPt-enplw&list=PLiEJsoZExlCyYwzGLQqwMTT8hvwfI5WYj

7 See http://www.facebook.com/terms.php

8
http://www.facebook.com/pages/The-National-Association-for-the-Advancement-of-White-People/102208269835141

9 On this issue, see Pole (1994, 2000).

10 Pioneer Fund, http://www.sourcewatch.org/index.php/Pioneer_Fund

11 SPLC, Jean-Philippe Rushton, https://www.splcenter.org/fighting-hate/extremist-files/individual/jean-philippe-rushton

12
http://cryptome.org/ricin-patent.pdf

13
*Brandenburg v Ohio,* 395 US 444 (1969), https://www.law.cornell.edu/supremecourt/text/395/444

14 For opposing argument articulating the importance of the First Amendment, see Baker (2009, 2012).
